# Potential of ezetimibe in memory deficits associated with dementia of Alzheimer's type in mice

**DOI:** 10.4103/0253-7613.59925

**Published:** 2009-12

**Authors:** Yogita Dalla, Nirmal Singh, Amteshwar Singh Jaggi, Dhandeep Singh, Pooja Ghulati

**Affiliations:** Department of Pharmaceutical Sciences and Drug Research, Punjabi University, Patiala, Punjab - 147 002, India; 1 Department of Swami Vivekanand College of Pharmacy, SVIET, Chandigarh Highway, Rajpura, India

**Keywords:** Alzheimer's disease, cholesterol, dementia, ezetimibe, memory

## Abstract

**Background:**

High cholesterol levels have been positively correlated with a higher incidence of memory impairment and dementia.

**Aim:**

The study was undertaken to investigate the potential of the lipid-lowering drug, ezetimibe, in memory deficits associated with dementia of Alzheimer's (AD) type in mice.

**Methods:**

Dementia was induced with chronic administration of a high-fat diet (HFD) or intracebroventricular streptozotocin (ICV STZ, two doses of 3 mg/kg) in separate groups of animals. The memory of the animals was assessed by employing a Morris water maze. Brain thio barbituric acid-reactive species and reduced glutathione levels were measured to assess the total oxidative stress. Brain acetyl cholinesterase (AChE) activity and total serum cholesterol levels were also measured.

**Results:**

STZ/HFD produced a significant impairment of memory along with an increase in brain AChE activity and oxidative stress. HFD mice also showed an increase in cholesterol levels. Ezetimibe (10 mg/kg, orally for 15 days) significantly attenuated STZ/HFD-induced memory deficits and biochemical changes. It also prevented HFD-induced rise in the cholesterol level.

**Conclusions:**

The memory-restorative effect of ezetimibe can be attributed to its cholesterol-dependent as well as cholesterol-independent effects. The study highlights the potential of ezetimibe in memory dysfunctions associated with dementia of AD.

## Introduction

Loss of memory, a prominent feature of old age, is associated with dementia of different types. Dementia is a syndrome of progressive nature marked by gross behavioral and personality disturbances. This syndrome occurs in Alzheimer's disease (AD), cerebrovascular disease (i.e., multi-infarct dementia) and other conditions primarily or secondarily affecting the brain. The dementia, which has gained much attention in the last decade, is dementia of AD. It is estimated that in 2010 over five million people will be diagnosed with probable AD in the United States alone. Increasing age is the greatest risk factor for AD; one-tenth of the elderly over 65 years of age develop AD, whereas nearly half of those over the age of 85 years are diagnosed with probable AD.[1] Clinically, AD is characterized by a gradual but progressive decline in memory and other cognitive domains and the frequent occurrence of non-cognitive behavioral symptoms. The main histological features of AD include extracellular deposition of ß-amyloid (Aß) plaques and intraneuronal neurofibrillary tangles. Loss of cortical cholinergic neurones in AD probably accounts for memory impairment.

Lipids and lipid peroxidation products play an important role in the homeostasis of the central nervous system. It has been reported that lipid transport genes and vascular changes associated with peripheral dyslipidemias increase the risk of AD.[2] Brain cholesterol is an essential component of neuronal cell membranes and is involved in a number of biological functions, such as membrane trafficking, signal transduction, myelin formation and synaptogenesis. Given these widespread activities, it is not surprising that dysfunctions in cholesterol synthesis, storage, transport and removal may lead to human brain diseases. Some of these diseases emerge as a consequence of genetic defects in the enzymes involved in cholesterol biosynthesis; in other cases, such as AD, there is a link between cholesterol metabolism and the formation and deposition of amyloid-beta peptide.[3]

Cholesterol turnover appears to play a crucial role in the deposition and clearance of amyloid peptide in the brain. Serum cholesterol, atherosclerosis, apolipoprotein-E and AD all appear to be interconnected.[3,4] ApoE is a cholesterol-transporting protein that is associated with amyloid deposits. Epidemiological studies have revealed that individuals with high peripheral cholesterol levels show more susceptibility to AD.[3–5] Moreover, experimental studies have shown that cholesterol-fed wild-type rabbits develop brain pathology similar to that of AD.[5] A transgenic mouse model of AD exhibited increased Aß plaques when mice were fed with a cholesterol-rich diet.[6] Cell culture and *in vivo* animal studies have shown that reducing cholesterol can inhibit Aß synthesis.[5,6] Degeneration that occurs in AD is mediated through the modulation of cholesterol signalling in the brain.[7] A high cholesterol level in mid-life is a risk for AD.[8] The National Institute of Health predicts that if the current trend continues, there will be more than 8.5 million AD patients by the year 2030 in the USA alone.

Therefore, a new approach aimed at controlling the blood cholesterol level is gathering momentum for the management of AD. Many experimental as well as clinical studies in the recent past have documented usefulness of cholesterol-lowering drug statins (HMG CoA reductase inhibitors) in memory dysfunction associated with AD and other dementias.[9] Epidemiological studies have suggested that individuals above the age of 50 years, who were receiving statins, had a substantially lowered risk of developing dementia.[10] In our previous studies, we have also reported the ameliorative effects of cholesterol-lowering drugs simvastatin, atorvastatin and pitavastatin in experimental amnesia.[11,12] Ezetimibe, a 2-azetidinone derivative, is a recently marketed cholesterol-lowering drug, which exerts its lipid-lowering action via selectively inhibiting the absorption of both dietary and biliary cholesterol from the intestine. Selective inhibition of intestinal cholesterol absorption provides an effective means to lower plasma cholesterol, which attributes to the potent lipid-lowering action of ezetimibe.[13] Although ezetimibe has gained much attention as a lipid-lowering drug, yet, it remains to be explored for its possible benefits in memory deficits associated with dementia. In forte of this therefore the present study has been undertaken to investigate the effect of ezetimibe in a high-fat diet (HFD)[11] as well as intracerebroventricular streptozotocin (ICV STZ)[12,14,15]-induced memory deficits of the AD type in mice.

## Materials and Methods

### Animals

Swiss albino mice, weighing 20–30 g, were employed in the present study (procured from CRI, Kausali, India). They were maintained on standard laboratory pellet chow diet (Kisan Feeds Ltd., Chandigarh, India) and water *ad libitum*. The animals were exposed to 12-h light and 12-h dark cycles. The mice were acclimatized to the laboratory conditions five days prior to the behavioural study. The experimental protocol was duly approved by the Institutional Animal Ethics Committee (IAEC) and care of the animals was performed as per the guidelines of the Committee for the Purpose of Control and Supervision of Experiments on Animals (CPCSEA), Ministry of Environment and Forest Government of India (Reg. No. CPCSEA/107/1999).

### Drugs and chemicals

Ezetimibe was obtained as a gift from Indswift Pharmaceuticals Ltd., Baddi (HP), India. Folin-Ciocalteu's Phenol reagent was purchased from Merck Limited, Mumbai, India. 5.5 dithiobis (2-nitro benzoic acid) (DTNB), reduced glutathione (GSH), bovine serum albumin (BSA) and thiobarbituric acid were obtained from Loba Chem, Mumbai, India. STZ and 1, 1, 3, 3-tetra methoxy propane were procured from Sigma-Aldrich, St. Louis, MO, USA. The cholesterol estimation kit was purchased from Monozyme India Ltd., Secundrabad, India. All the reagents used in this study were of analytical grade. Ezetimibe was suspended in 0.5% w/v sodium carboxymethyl cellulose (CMC). STZ was dissolved in artificial cerebrospinal fluid (ACSF) prepared according to the method as described in our earlier study.[15] Ezetimibe and CMC were administered orally with the help of an oral tube (canulla) and STZ and ACSF were delivered intracerebroventricularly.

## Laboratory Models

### Exteroceptive behavioral model

Morris water maze (MWM) test was employed to assess learning and memory of the animals.[16] MWM is a swimming-based model where the animal learns to escape on to a hidden platform. It consists of a large circular pool (150 cm in diameter, 45 cm in height, filled to a depth of 30 cm with water at 28 ± 1°C). The water was made opaque with white-colored non-toxic dye. The tank was divided into four equal quadrants with the help of two threads fixed at right angles to each other on the rim of the pool. A submerged platform (10 cm^2^), painted in white, was placed inside the target quadrants of this pool, 1 cm below surface of the water. The position of the platform was kept unaltered throughout the training session. Each animal was subjected to four consecutive training trials on each day with an intertrial gap of 5 min. The mouse was gently placed in the water between the quadrants, facing the wall of the pool, with the drop location changing for each trial, and allowed 120 s to locate the submerged platform. Then, it was allowed to stay on the platform for 20 s. If it failed to find the platform within 120 s, it was gently guided onto the platform and allowed to remain there for 20 s. The day 4 escape latency time (ELT) to locate the hidden platform in the water maze was noted as index of acquisition or learning. The animal was subjected to training trials for four consecutive days, the starting poison was changed with each exposure as mentioned below and the target quadrant (Q 4) remained constant throughout the training period.

**Table:** 

Day1	Q1	Q2	Q3	Q4
Day2	Q2	Q3	Q4	Q1
Day3	Q3	Q4	Q1	Q2
Day4	Q4	Q1	Q2	Q3

On the fifth day, the platform was removed and each mouse was allowed to explore the pool for 120 s. The mean time spent in all the four quadrants was noted. The mean time spent by the animal in the target quadrant searching for the hidden platform was noted as index of retrieval.

The experimenter always stood at the same position. Care was taken that the relative location of the water maze with respect to other objects in the laboratory serving, as prominent visual clues, were not disturbed during the total duration of the study. All the trials were completed between 09.00 and 17.00 h.

### Interoceptive behavioral models

- STZ-induced dementia.
- HFD-induced dementia.

## ICV administration of STZ

Mice were anesthetised with anesthetic ether for intracerebroventricular (ICV) administrations. Ether has been prefered here due to its ultrashort action and fast reversibility. Moreover, a brief extent of ether exposure for ICV injection has been reported to exert no significant effect on the learning and memory behavior of the animals.[14,15,17] ICV injections were made with a hypodermic neddle of 0.4 mm external diameter attached to a 10 *μ*l Hamilton microlitre syringe (Top Syringe, Mumbai, India). The needle was covered with a polypropylene tube except 3 mm of the tip region so as to insert this portion of the needle perpendicularly through the skull into the brain of the mouse. The injection site was 1 mm to the right or left midpoint on the line drawn through to the anterior base of the ears. Injections were performed into the right or left ventricle randomly. Two doses of STZ (3 mg)kg) were administered (by ICV injection bilaterally), the second dose being administered after 48 h of the first dose. The concentration was adjusted so as to deliver 10 *μ*L in an injection. The injection was made at two places as it is difficult to administered 10 *μ*L at a single site. The control group mice were given an ICV injection of ACSF in a similar manner.

## HFD-induced dementia

Animals were subjected to a cholesterol-rich diet (HFD) for 90 days to induce memory impairment[11] [Table 1].

**Table 1:** Composition of high-fat diet

*Feed contents*	*Weight (g)*
Powdered pellet diet (standard laboratory diet)	365
Lard	310
Casein	250
Cholesterol	10
Sodium cholate	5
DL-Methionine	3
Yee-sac powder	1
*Vitamins and minerals	55
Sodium chloride	1
Total	1000

* Composition of vitamins and minerals per kg of HFD. Vitamin A (120,000 I.U.), vitamin D3 (24,000 IU), vitamin B2 (48 mg), vitamin E (184 units), vitamin K (24 units), calcium pantothenate (60 mg), nicotinamide (240 mg), vitamin B12 (144 mg), calcium (18 mg), magnesium (660 mg), iodine (24 mg), iron (180 mg), zinc (360 mg), copper (48 mg), cobalt (108 mg)

## Collection of samples

Animals were sacrificed by cervical dislocation and the brains were removed and homogenized in phosphate buffer (pH = 7.4). The homogenates were then centrifuged at 3000 rpm for 15 min. The supernatant of the homogenate was used for biochemical estimation as per the following methods.

## Estimation of brain acetyl cholinesterase (AChE) activity

The whole brain AChE activity was measured by the method of Ellman *et al*.[18] with slight modifications.[12] Change in absorbance per min of the sample was read spectrophotometrically (DU 640B spectrophotometer, Beckman Coulter Inc., Fullerton, CA, USA) at 420 nm.

## Estimation of brain thiobarbituric acid-reactive species (TBARS) level

The whole brain TBARS level was measured by the method of Ohokawa *et al.*[19] with slight modifications. The absorbance was measured spectrophotometrically at 532 nm.

## Estimation of brain GSH level

The whole brain GSH level was measured by the method of Beutler *et al.*[20] with slight modifications. The absorbance was measured spectrophotometrically at 412 nm.

## Estimation of brain total protein

For the estimation of total protein in the brain, the method of Lowry *et al.*[21] with slight modifications was used. The absorbance was determined spectrophotometrically at 750 nm.

## Estimation of total cholesterol

The total serum cholesterol levels were estimated by Allain's method by employing the commercially available standard cholesterol estimation kit. The absorbance was measured against a blank at 540 nm spectrophotometrically.

## Experimental Protocol

Eight groups of mice were employed in the present study and each group comprised of 10 mice.

### Group I (control)

Mice were administered distilled water (10 ml/kg p.o.) 30 min before conducting acquisition trials from day 1 to day 4 and 30 min before the retrieval trial conducted on day 5.

### Group II (CMC control)

Mice were administered 0.5% w/v CMC, (10 ml/kg p.o.) daily for 15 days and then subjected to the MWM test. The vehicle was also administered 45 min before the acquisition trial conducted from day 1 to day 4 and before the retrieval trial conducted on day 5.

### Group III (Ezetimibe per se)

Mice were administered ezetimibe (10 mg/kg, p.o.) suspended in CMC (0.5 % w/v) for 15 days and then subjected to the MWM test. The treatment was continued (administered 45 min before) during the acquisition trial conducted from day 1 to day 4. The animals were administered vehicle (0.5% w/v CMC, 10 ml/kg, p.o.) 45 min before the retrieval trial conducted on day 5.

### Group IV (HFD control)

Mice were put on a HFD for 90 days followed by exposure to the MWM test.

### Group V (HFD + ezetimibe)

HFD mice were treated with ezetimibe (10 mg/kg/day, p.o.) for 15 days and then subjected to the MWM test. The administration of ezetimibe was continued (administered 45 min before) during the acquisition trial conducted from day 1 to day 4. The animals were administered vehicle (0.5% w/v CMC, 10 ml/kg, p.o.) 45 min before the retrieval trial conducted on day 5.

### Group VI (ACSF control)

Mice were injected ICV ACSF (25mg/ml, 10 *μ*l) in two dosage schedules, i.e. on the first and third days followed by exposure to the MWM test after 15 days.

### Group VII (STZ control)

Mice were injected ICV STZ (3 mg/kg, 10 *μ*l) in two dosage schedules, i.e. on the first and third days followed by exposure to the MWM test after 15 days.

### Group VIII (ICV STZ + ezetimibe)

ICV STZ mice were treated with ezetimibe (10 mg/kg, p.o.) for 15 days (starting after the second dose of STZ) and then subjected to the MWM test. The administration of ezetimibe was continued (administered 45 min before) during the acquisition trial conducted from day 1 to day 4. The animals were administered vehicle (0.5% w/v CMC, 10 ml/kg, p.o.) 45 min before the retrieval trial conducted on day 5.

### Statistical analysis

The results were expressed as mean ± standard error of means (SEM). The data obtained from various groups were statistically analyzed using one-way ANOVA followed by Tukey's Multiple Range test. A *P-* value <0.05 was considered to be statistically significant.

## Results

### Effect of HFD/STZ on ELT, and time spent in target quadrant (TSTQ) using MWM

**Figure 1:**
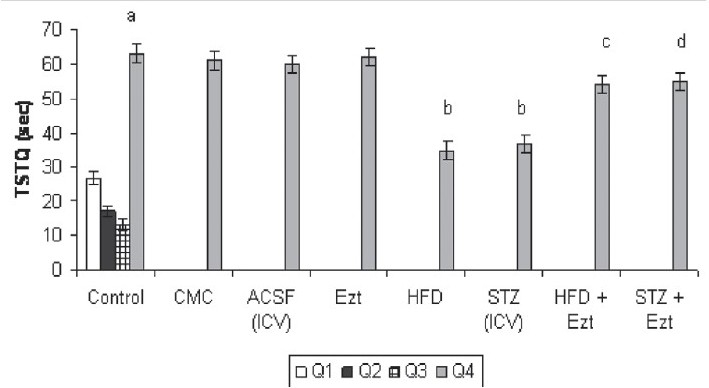
Effect of ezetimibe on high-fat diet (HFD) and streptozotocin (STZ)-induced changes in time spent in target quadrant (TSTQ) using Morris water maze. CMC, carboxymethylcellulose; ACSF, artificial cerebrospinal fluid; Ezt, ezetimibe Each group (n = 10) represents mean ± SEM ^a^*P* < 0.05 vs. time spent in the other quadrants of control ^b^*P* < 0.05 vs. TSTQ of control group ^c^*P* < 0.05 vs. TSTQ of the HFD group ^d^*P* < 0.05 vs. TSTQ of STZ mice

Distilled water, CMC and artificial CSF-treated mice showed a downward trend in their ELT on subsequent exposure to MWM. There was a significant fall in day 4 ELT when compared to day 1 ELT of these mice, reflecting normal learning ability [Table 2]. Further, these animals showed a significant rise in day 5 TSTQ when compared to time spent in other quadrants during the retrieval trial conducted on day 5 therefore reflecting normal retrieval (memory) as well [Figure 1].

**Table 2:** Effect of ezetimibe on HFD and STZ-induced changes in the ELT of mice using MWM

*Group*	*Treatment*	*Dose (kg^-1^)*	*ELT (day 1) in s*	*ELT (day 4) in s*
I	Control	10 ml p.o.	98.2 ± 3.6	40.4 ± 2.1^a^
II	CMC control	10 ml p.o.	95.4 ± 3.8	38.5 ± 2.3
III	Ezt	10 mg p.o.	96 ± 4.7	39 ± 2.5
IV	HFD treated for 90 days		99 ± 4.4	78.1 ± 2.6^b^
V	HFD + Ezt	HFD + 10 mg p.o.	97 ± 7.5	55 ± 2.4^c^
VI	ASCF control	10 μl ICV	98.4 ± 3.4	43.3 ± 2.7
VII	STZ	3 mg ICV	100.2 ± 4.8	80.2 ± 2.4^b^
VIII	STZ + Ezt	3 mg ICV + 10 mg p.o.	99 ± 4.3	60 ± 3.5^d^

CMC, carboxymethylcellulose; ACSF, artificial cerebrospinal fluid; Ezt, ezetimibe, Each group (n = 7) represents mean ± SEM

a *P* < 0.05 as compared to 1^st^ day ELT in control

b *P* < 0.05 as compared to day 4 ELT in control

c *P* < 0.05 as compared to day 4 ELT in the HFD group

d *P* < 0.05 as compared to day 4 ELT in STZ mice

STZ-treated mice and mice subjected to HFD showed a significant increase in day 4 ELT when compared to day 4 ELT of the respective control, indicating impairment of acquisition [Table 2]. Further, a significant decrease in day 5 TSTQ of these animals was also noted, reflecting impairment of memory as well [Figure 1].

### Effect of ezetimibe on HFD/STZ-induced impairment of learning and memory using MWM

Administration of ezetimibe significantly attenuated HFD-induced rise in day 4 ELT [Table 2] along with reversal of HFD-induced fall in day 5 TSTQ, indicating reversal of HFD-induced learning and memory deficits [Figure 1]. Treatment of ezetimibe also prevented STZ-induced rise in day 4 ELT [Table 2] and fall in day 5 TSTQ [Figure 1]. However, administration of ezetimibe *per se* did not show any significant per se effect on day 4 ELT [Table 2] and TSTQ [Figure 1].

### Effect of ezetimibe on HFD and STZ-induced changes in brain AChE activity

**Figure 2:**
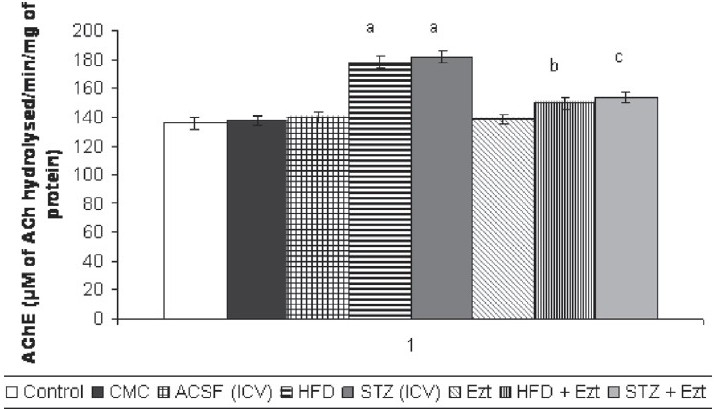
Effect of ezetimibe on high-fat diet (HFD)/streptozotocin (STZ) induced changes in brain acetylcholinesterase (AChE) activity. CMC, carboxymethylcellulose; ACSF, artificial cerebrospinal fluid; Ezt, ezetimibe Each group (n = 10) represents mean ± SEM ^a^*P* < 0.05 vs. brain AChE activity of control ^b^*P* < 0.05 vs. brain AChE activity of the HFD-treated group ^c^*P* < 0.05 vs. brain AChE activity of STZ-treated mice

HFD for 90 days/STZ treatment showed a significant increase in brain AChE activity of mice when compared with their respective controls [Figure 2]. Treatment with ezetimibe significantly prevented HFD/STZ-induced rise in brain AChE activity [Figure 2]. However, ezetimibe per se did not show any *per se* effect on the brain AChE activity [Figure 2].

### Effect of ezetimibe on HFD and STZ-induced changes in the oxidative stress levels of the brain

**Figure 3:**
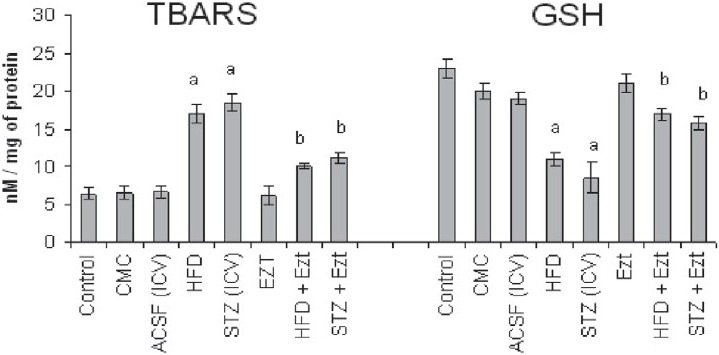
Effect of ezetimibe on high-fat diet (HFD)/streptozotocin (STZ) induced changes in brain thiobarbituric acid reactive species (TBARS) and reduced glutathione (GSH) levels. CMC, carboxymethylcellulose; ACSF, artificial cerebrospinal fluid; Ezt, ezetimibe Each group (n = 10) represents mean ± SEM ^a^*P* < 0.05 vs. control ^b^*P* < 0.05 vs. HFD-treated group ^c^*P* < 0.05 vs. STZ-treated mice

HFD for 90 days/STZ treatment showed a significant increase in the oxidative stress levels of the brain manifested in terms of increased TBARS level [Figure 3] and a decrease in the level of reduced form of glutathione (GSH) [Figure 3] when compared with their respective controls. Treatment with ezetimibe significantly prevented HFD/STZ-induced rise in brain oxidative stress [Figure 3]. However, ezetimibe *per se* did not show any effect on the brain oxidative stress levels [Figure 3].

### Effect of ezetimibe on HFD-induced changes in total serum cholesterol levels

**Figure 4:**
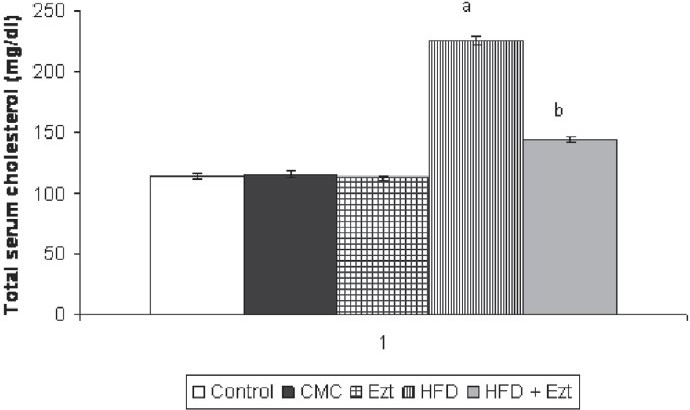
Effect of ezetimibe on high-fat diet (HFD) induced changes in serum total cholesterol level. CMC, carboxymethylcellulose; ACSF, artificial cerebrospinal fluid; Ezt, ezetimibe Each group (n = 10) represents the mean ± SEM ^a^*P* < 0.05 vs. total serum cholesterol of control ^b^*P* < 0.05 vs. total serum cholesterol of the HFD-treated group

Mice subjected to a HFD for 90 days showed a significant increase in the total serum cholesterol levels of the animals when compared with the control group [Figure 4]. Treatment with ezetimibe attenuated the HFD-induced rise in total serum cholesterol levels [Figure 4]. However, ezetimibe *per se* did not show any significant effect on the total serum cholesterol levels [Figure 4].

## Discussion

MWM employed in the present investigation is one of the most widely accepted models to assess learning and memory in rodents.[16] In water maze, a significant decrease in day 4 ELT of control animals during acquisition trials denoted normal acquisition of memory and an increase in the TSTQ in search of the missing platform during the retrieval trial conducted on day 5, indicating retrieval of memory. These results are consistent with our earlier findings.[11,12] No per se effects of vehicles employed in the present study to prepare various solutions of drugs were observed on acquisition and retrieval of memory. Therefore, the effect of pharmacological interventions on acquisition and retrieval of memory can be attributed to them and to their vehicles.

In the present study, chronic (90 days) administration of HFD not only produced a significant rise in total serum cholesterol levels but also impaired learning and memory. A significant enhancement of brain AChE activity and brain oxidative stress (increase in TBARS and decrease in GSH) levels were also observed. Clinical studies have suggested that the net brain cholesterol concentration is regulated by the serum cholesterol level and there is a cross-talk between the CNS and peripheral cholesterol pools.[22] Therefore, it is plausible that peripheral cholesterol levels modulate CNS cholesterol levels and vice versa. Elevated serum cholesterol levels not only lead to atherosclerosis but also carry a high risk of developing AD.[23] Epidemiological studies revealed that individuals with high peripheral cholesterol levels show more susceptibility to AD and the incidence of AD is higher in countries with high-fat and high-calorie diets.[24] It has been reported that rats fed with a special diet having a higher amount of fats showed memory deficits.[25] Therefore, HFD-induced memory deficits noted in the present study closely mimic the clinical manifestations of AD, i.e. cognitive decline, and highlight the importance of cholesterol in the pathophysiology of AD. Administration of ezetimibe to HFD mice produced a significant reversal of elevated serum cholesterol levels and memory impairment. In addition, ezetimibe also prevented HFD-induced rise in the brain oxidative stress level and brain AChE activity. Because HFD-induced cognitive deficits can be overcome by cholesterol lowering drugs,[11] the cholesterol lowering property appears to be primarily responsible for the observed reversal of HFD-induced memory impairment by ezetimibe. Further, ezetimibe recently has been shown to exert neuroprotective,[26] antioxidant[27] as well as antiamyloid[28] actions. Hence, these cholesterol-independent actions of ezetimibe can also not be ignored at this point, which probably explains the attenuation of HFD-induced rise in brain oxidative stress (increased TBARS and reduced GSH) and brain AChE activity.

In our study, ICV STZ significantly impaired learning and memory in mice. The ICV STZ model has been described as an appropriate animal model for AD.[14] After ICV STZ administration, a progressive decline in memory functions has been observed, which is a characteristic feature of dementia of AD. Although the mechanism of action of STZ on memory impairment is not yet known, it probably involves the induction of oxidative stress[29] to which myelin is particularly vulnerable. Damage to myelin by oxidative stress is seen in disorders such as AD with cognitive impairment.[30] In the present study, treatment with ezetimibe significantly improved STZ-induced memory deficits along with attenuation of STZ-induced rise in brain AChE activity and brain oxidative stress levels. As mentioned above, few recent reports have documented the potential antioxidative[27] and neuroprotective[26] actions of ezetimibe. Our results support this contention, whereby ezetimibe has significantly reversed STZ-induced rise in brain oxidative stress levels and brain AChE activity. Therefore, the potential neuroprotective and antioxidative (cholesterol independent) actions of ezetimibe may be speculated to improve memory dysfunctions associated with ICV STZ in this study.

On the basis of the above discussion, it may be concluded that ezetimibe reversed HFD and ICV STZ-induced memory deficits by virtue of its cholesterol-dependent as well as cholesterol-independent actions. Perhaps this is the first report implicating the memory-restorative potential of ezetimibe in experimental dementia of the AD type.
